# Bibliometric Analysis Reveals a 20-Year Research Trend for Chemotherapy-Induced Peripheral Neuropathy

**DOI:** 10.3389/fneur.2021.793663

**Published:** 2022-02-08

**Authors:** Jialin Gu, Miao Hu, Zhancheng Gu, Jialin Yu, Yi Ji, Lingchang Li, Canhong Hu, Guoli Wei, Jiege Huo

**Affiliations:** ^1^Department of Oncology, Affiliated Hospital of Integrated Traditional Chinese and Western Medicine, Nanjing University of Chinese Medicine, Nanjing, China; ^2^The Third Clinical Medical College, Nanjing University of Chinese Medicine, Nanjing, China; ^3^Department of Oncology, Jiangsu Province Academy of Traditional Chinese Medicine, Nanjing, China; ^4^Department of Oncology, Nanjing Lishui District Hospital of Traditional Chinese Medicine, Nanjing, China; ^5^Department of Oncology, Yangzhou University Medical College, Yangzhou, China

**Keywords:** chemotherapy-induced peripheral neuropathy, bibliometric analysis, research hotspots, publications, VOSviewer

## Abstract

**Objective:**

A lot of research has focused on the field of chemotherapy-induced peripheral neuropathy (CIPN). In this study, we performed a bibliometric analysis of CIPN-related publications to identify the key research areas and trends over the last 20 years.

**Methods:**

We searched the Web of Science core collection for publications related to CIPN that were published between January 2001 and September 2021. We then performed bibliometric analysis and visualization using Microsoft Excel 2019, VOSviewer, and the Bibliometric online analysis platform (https://bibliometric.com/).

**Results:**

In total, we identified 2,188 eligible publications in the field of CIPN, with an increasing trend in the annual number of publications. The United States and Italy were dominant in the CIPN field. *Supportive Care in Cancer* was the most productive journal. G. Cavaletti and A.A. Argyriou published the largest number of papers. Of all institutions, the University of Milano-Bicocca, Italy, published the highest number of papers. Analysis of the co-occurrence of keywords revealed the specific characteristics relating to the four main clusters: oxaliplatin, paclitaxel, pain management, and quality of life (QOL). Newly emerging research focusses predominantly on neuroinflammatory mechanisms and non-pharmacological interventions for CIPN.

**Conclusion:**

This bibliometric study reviewed the evolutionary trends in CIPN research and identified current research hotspots and research trends. In addition, we identified journals, institutions, and authors, with the highest levels of impact to enhance the collaboration and learning.

## Introduction

Chemotherapy-induced peripheral neuropathy (CIPN) has become a rising health concern due to the increasing number of cancer survivors and prolonged survival rates. CIPN is a prevalent clinical problem in over 50% of patients treated with neurotoxic medications and undoubtedly increases the burden on patients and healthcare coverage (1, 2). Approximately 30% of patients still report varying degrees of CIPN 6 months after chemotherapy; this condition has a notable effect on the lives in cancer survivors (3, 4). A previous study on the ovarian cancer showed that the symptoms of CIPN did not diminish until 3 years after the end of treatment (5). At present, the mechanistic studies predominantly focus on the dorsal root ganglion injury, microtubule injury, mitochondrial dysfunction, oxidative stress, neuroinflammation, and the dysfunction of membrane channels (6, 7). There is no effective strategy for preventing CIPN and no recognized therapeutic agent at the present time. Only duloxetine is recommended for the chemotherapy-induced neuropathic pain (8, 9). Over the past two decades, thousands of published manuscripts have reported mechanistic, diagnostic, therapeutic, and preventive studies of CIPN, making it very difficult for researchers to identify highly influential articles and research hotpots. Manual literature searches can no longer accurately identify potential trends in the development of CIPN. Therefore, there is an urgent need to develop new literature retrieval strategies for the fast data collation and mining.

Bibliometric analysis is a statistical method used for the analysis and visualization of key characteristics from published articles and the identification of research trends in a specific field using online literature databases (10, 11). Bibliometric analysis provides us with tools to analyze the publications qualitatively and quantitatively and identify significant research hotspots and trends. In addition, bibliometric analysis can accurately identify the most influential researchers, manuscripts, journals, and institutions (12). Bibliometrics has been applied widely in a variety of medical fields, such as complementary and alternative medicine (13), oncology (14), infectious diseases (15), nursing (16), and encephalopathy (17). Although there is a significant body of literature available for CIPN, these publications have not been summarized and analyzed. Therefore, in the present study, we conducted a comprehensive bibliometric analysis of publications related to CIPN from 2001 to 2021. Our goal was to gain enhanced understanding of existing research hotspots and potential trends and provide useful reference guidelines for future researchers.

## Materials and Methods

### Data Selection

**Figure 1:**
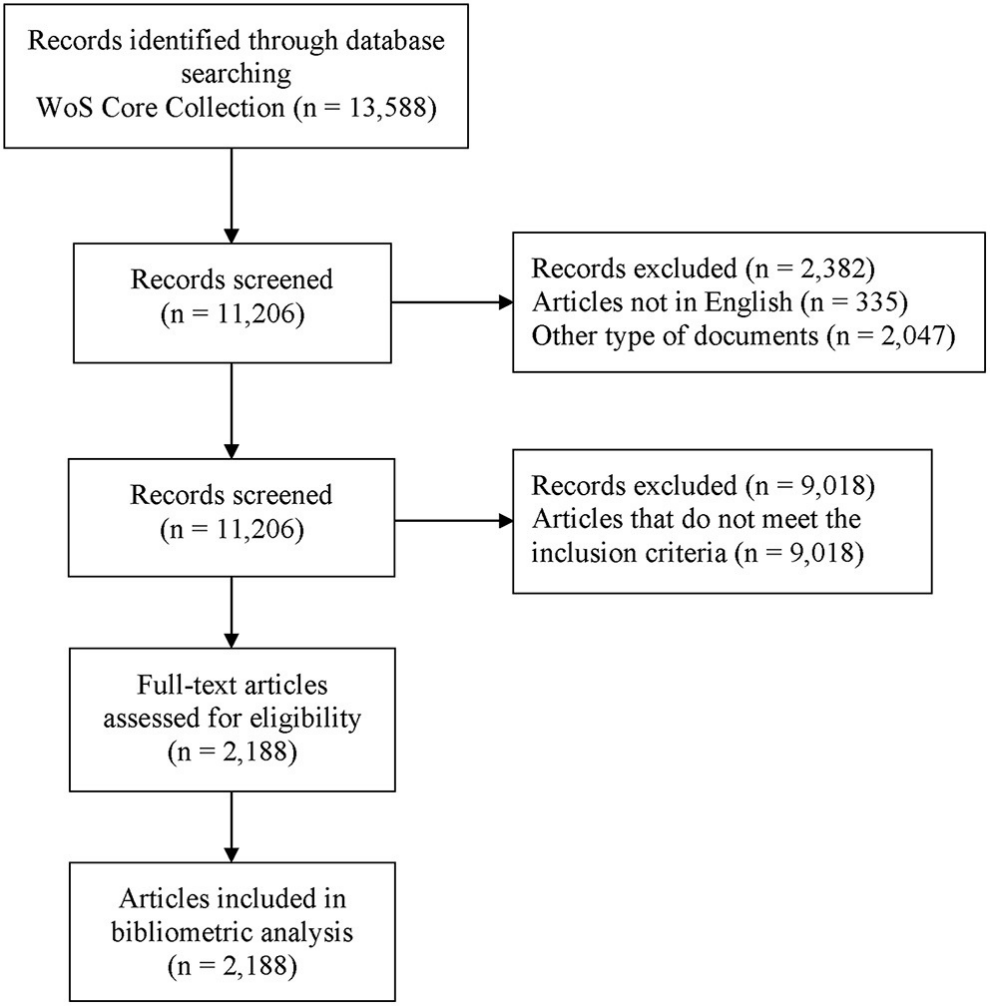
Flowchart of study identification and selection.

We screened the Web of Science Core Collection (WoSCC, Clarivate Analytics) between January 2001 and September 2021 to identify publications related to CIPN. The details of search terms are shown in Supplementary Table 1. There was no limit to the word term “peripheral”; this strategy aimed to avoid omissions as much as possible. Only papers published in English were considered. The title and abstract were reviewed and screened by two independent reviewers; a third reviewer decided on any disagreements. Figure 1 shows the flow chart of study identification and selection.

### Data Analysis and Visualization

A range of key information was extracted from the articles that met the required criteria, such as title, author, research institution, country or region, keywords, year of publication, source, number of citations, 2020 impact factor (IF), and Hirsch index (H-index). All records were downloaded from the WoSCC for further analysis. Microsoft Excel 2019, VOSviewer (Version 1.6.15, Leiden University, The Netherlands), the Bibliometric online analysis platform (https://bibliometric.com/), and the biblioshiny package from R language (18) were used for data processing and visualization of the above variables. The H-index was defined as the number of papers published and cited ≥ h; thus, helped to identify the high-quality output from given researchers (19). VOSviewer is a scientific software package for the bibliometric analysis and visualization (20).

## Results

### Publication Characteristics

**Figure 2:**
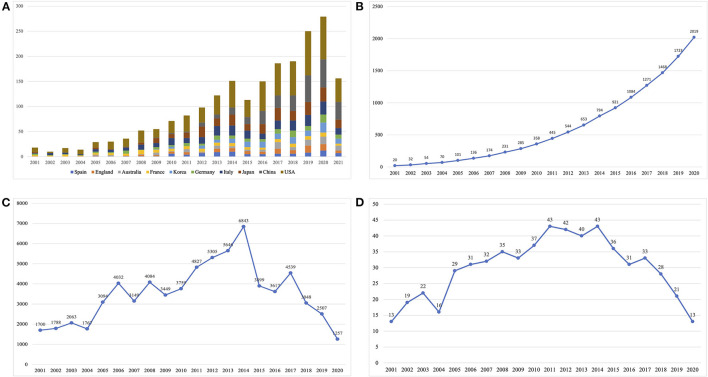
The number of publications, the number of citations, and the Hirsch index (H-index) for chemotherapy-induced peripheral neuropathy (CIPN): **(A)** the annual number of publications in major countries; **(B)** the annual cumulative number of publications; **(C)** the annual citation number of publications, and **(D)** the annual H-index of publications.

Literature searches identified a total of 13,588 publications from the WoSCC. Based on the titles and abstracts of all articles screened by the reviewers, 2,188 studies met the inclusion criteria and were included for further data extraction that included 1,899 articles (86.7%) and 289 reviews (14.3%). Figure 1 summarizes the research characteristics of these publications. A growing trend in publication number was observed, indicating the increasing attention and interest in the CIPN field (Figures 2A,B). In addition, we constructed publication trends for the CIPN field using Microsoft Excel 2019 (*y* = 0.0373*x*^3^ – 0.3597*x*^2^ + 6.2327*x* + 4.782, *R*^2^ = 0.9876); these analyses indicated that there will be more than 500 publications related to CIPN by 2025. Additionally, we found that the number of citations increased rapidly after 2005 and reached the highest level in 2014 (Figure 2C). Overall, 170 studies were cited more than 100 times. The average H-index in this field was 30, peaking in 2011 and 2014 (Figure 2D). Collectively, these data showed that significant progress had been achieved in the CIPN field over the last decade, particularly between 2011 and 2014.

### The Distribution of Publications by Country

**Figure 3:**
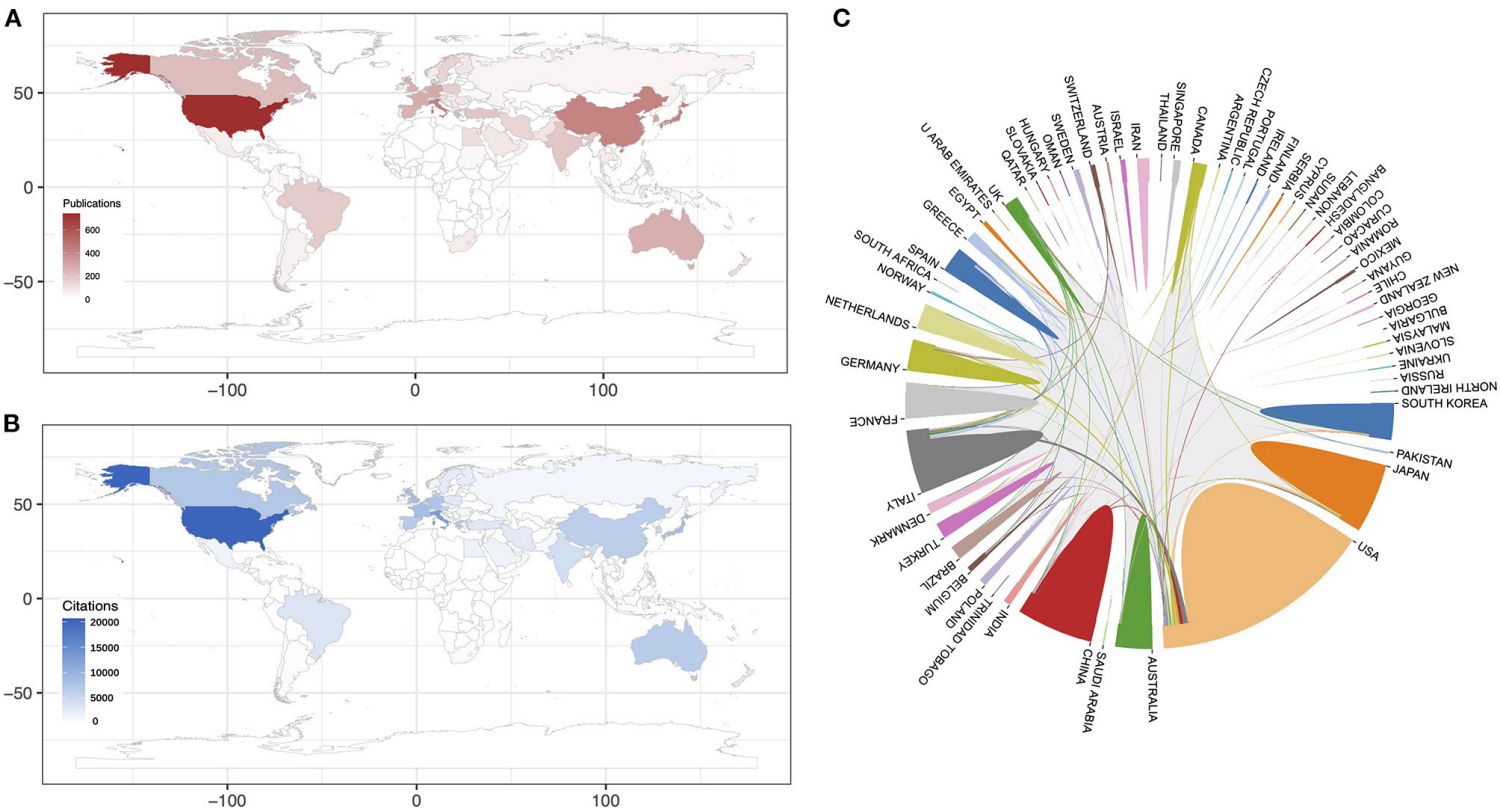
Countries associated with the CIPN field between 2001 and 2021: **(A)** the number of publications arising from various countries; **(B)** the total number of citations from various countries, and **(C)** international collaboration between countries.

A total of 69 countries and regions were involved in the publication of the 2,188 articles and reviews. The United States contributed the most significant number of publications (740), followed by Italy (247), Japan (246), China (243), and Germany (116) (Figure 3A). The United States (29, 866), Italy (11,371), and Japan (4,961), were associated with the highest number of citations, thus showing the comparative breadth of these countries in this field (Figure 3B). Despite the small number of publications, France, and the Netherlands had more citations on average than the most countries, indicating the high quality of the studies carried out in these countries. Close research cooperation between countries is known to drive technological and research advances. Figure 3C shows the cooperation between countries in the CIPN field. We found closer cooperation among the United States, Italy, Germany, and other countries, while China and Japan were associated with lower levels of international cooperation.

### The Distribution of Publications by Institution

Table 1 shows the top 10 institutions in terms of publication number, total citations, and the average number of citations. The University of Milano-Bicocca, the University of Michigan, the Mayo Clinic, and the MD Anderson Cancer Center had the most vital partnerships with other institutions.

**Table 1:** The top 10 institution in terms of publications in the chemotherapy-induced peripheral neuropathy (CIPN) field.

**Institution**	**Country**	**Publications**	**Total citations**	**Average citation**
University of Milano-Bicocca	Italy	117	5,976	51.08
Mayo Clinic	USA	82	5,075	61.89
MD Anderson Cancer Center	USA	82	3,273	39.91
University of Michigan	USA	65	2,596	39.94
University of Sydney	USA	45	965	21.44
Johns Hopkins University	USA	43	1,306	30.37
University of California, San Francisco	USA	43	1,361	31.65
University of Florence	USA	39	1,457	37.36
Dana-Farber Cancer Institute	USA	36	1,940	53.89
Kyung Hee University	Korea	34	417	12.26

### Author Distribution

We identified the 15 most influential authors in the CIPN field based on the H-index and the number of publications (Table 2). Scholars from Italy (5/15) made an enormous contribution to the CIPN field. G. Cavaletti had the highest H-index, number of publications, and total citations. Notably, although two Greek researchers were described as the most influential in terms of publications, Greek institutions are not included in Table 1, which we believe is mainly because the same author marked different institutions in different papers. In addition, we analyzed the collaborative relationships between highly productive authors in this field (Supplementary Figure 1). Based on a partnership map, we identified several key research teams, such as those of G. Cavaletti, S. B. Park, P. M. Dougherty, and C. I. Loprinzi; all these authors had close collaborative relationships with each other.

**Table 2:** The top 15 most influential authors.

**Author**	**Country**	**Articles *(n)***	**Total citations**	**Average citations**	**H-index**
Cavaletti G	Italy	113	3,511	31.07	44
Argyriou AA	Greece	48	1,566	32.63	26
Park SB	England	46	887	19.28	17
Alberti P	Italy	42	941	22.40	20
Bruna J	Spain	41	973	23.73	22
Loprinzi CI	USA	39	1,423	36.49	25
Goldstein D	Australia	38	918	24.16	16
Smith EML	USA	38	1,088	28.63	15
Kalofonos HP	Greece	37	1,304	35.24	25
Dougherty PM	USA	36	1,249	34.69	27
Ghelardini C	Italy	35	405	11.57	18
Marmiroli P	Italy	31	973	31.39	19
Mannelli LD	Italy	30	358	11.93	17
Egashira N	Japan	29	433	14.93	15
Velasco R	Spain	29	656	22.62	17

### Journal Distribution

Analysis showed that a total of 544 journals were involved in CIPN research during our study period. Table 3 shows the top 15 most productive journals in the CIPN field. Over the past 20 years, *Supportive Care in Cancer* had the highest number of publications; this was followed by *Pain* and *Molecular Pain*. *PAIN, Supportive Care in Cancer*, and the *Journal of Clinical Oncology*, were the top three journals with the most citations. Notably, the *Journal of Clinical Oncology* had the highest average number of citations; this indicated the higher quality of manuscripts published in this journal.

**Table 3:** The top 15 most productive journals between 2001 and 2021.

**Journal**	**No. of texts**	**Citations**	**Average citations**	**H-index**	**IF (2020)**	**5-year IF**
Supportive Care in Cancer	107	3,029	28.31	31	3.603	3.958
PAIN	56	4,099	73.20	32	6.961	7.704
Molecular Pain	40	1,613	40.33	22	3.395	3.615
Experimental Neurology	39	2,060	52.82	21	5.33	5.34
Journal of the Peripheral Nervous System	38	1,378	36.26	19	3.494	3.455
PLoS One	35	1,160	33.14	17	3.24	3.788
Cancer Chemotherapy and Pharmacology	34	785	23.09	16	3.333	3.402
Clinical Cancer Research	30	1,778	59.27	23	12.531	12.836
Scientific Reports	30	387	12.90	12	4.379	5.133
International Journal of Molecular Sciences	27	234	8.67	7	5.923	6.132
Neuroscience Letters	26	839	32.27	13	3.036	2.855
European Journal of Cancer	25	2,070	82.80	20	9.162	9.305
Journal of Pain	24	1,391	57.96	15	5.820	7.061
Journal of Pharmacological Sciences	24	336	14.00	11	3.337	3.432
Neuropharmacology	24	671	27.96	16	5.25	5.283

### Analysis of Hotspots and Trends in Research

**Figure 4:**
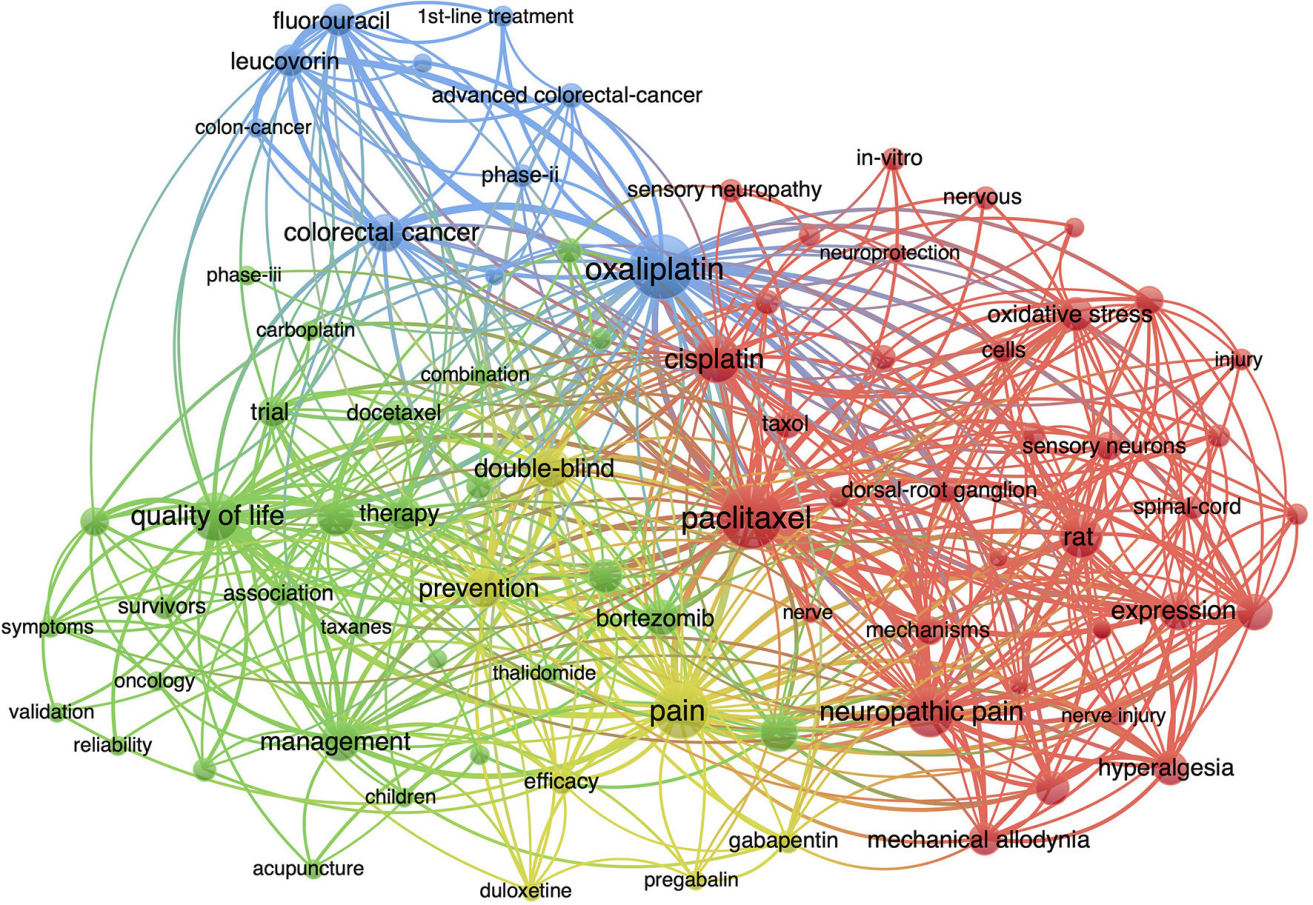
Visualization of keywords related to CIPN research between 2000 and 2021.

The distribution of co-occurring keywords and keywords plus were analyzed using VOSviewer to identify key trends and directions in the CIPN field. Descriptive keywords such as “peripheral neuropathy,” “neurotoxicity,” “cancer,” and “chemotherapy” were excluded. Keywords with frequencies >40 were analyzed; this analysis identified 80 keywords with the highest relevance (Figure 4). These keywords were divided into four clusters: (i) oxaliplatin, (ii) paclitaxel, (iii) pain management, (iv) and quality of life (QOL).

**Figure 5:**
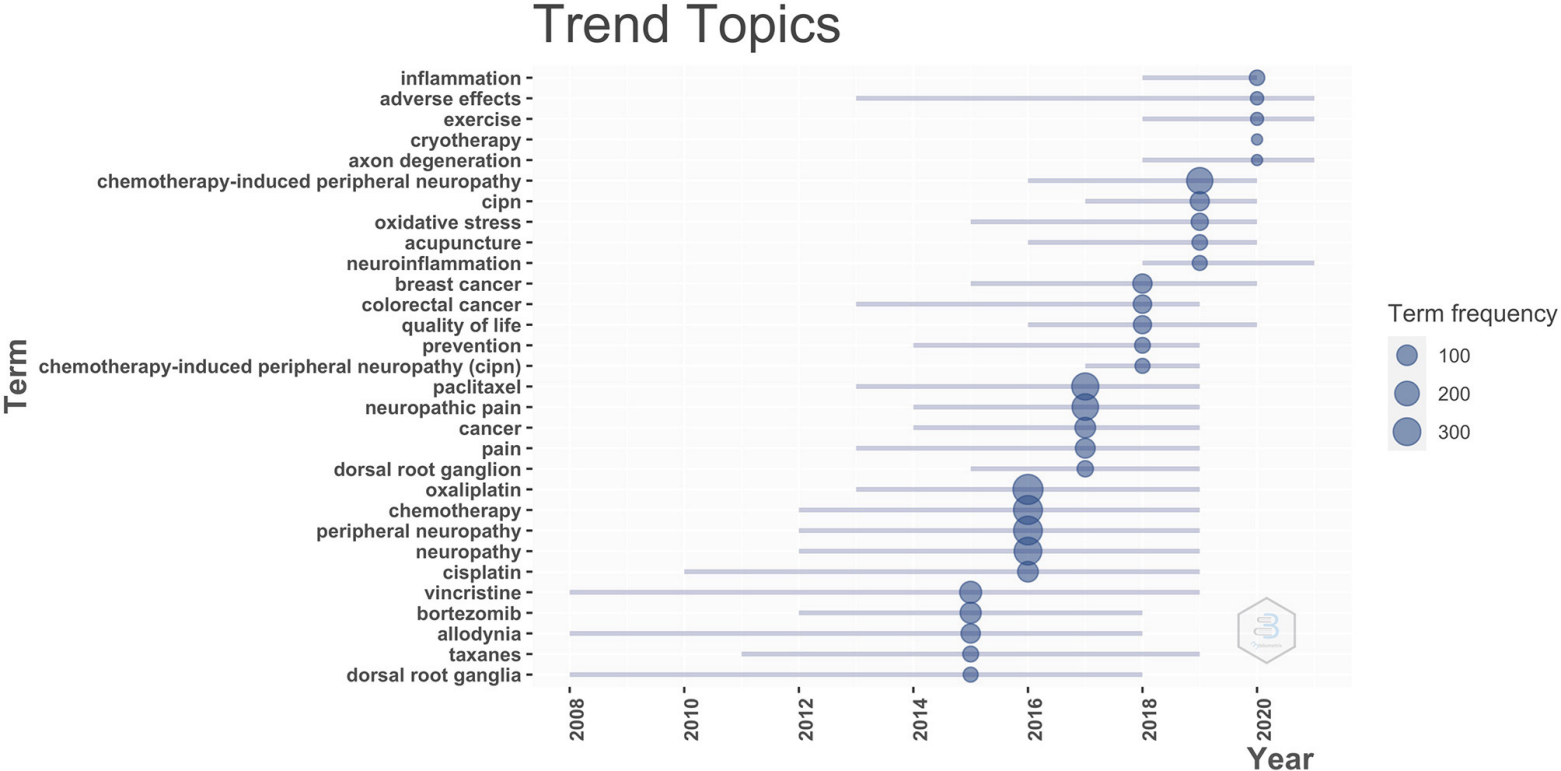
Timeline of research trends in the field of CIPN.

Next, we identified research trends in the field of CIPN so that these could be used as a reference for future researchers. We analyzed and visualized the temporal trends of keyword occurrences (Figure 5). Emerging trends in research on CIPN over the most recent 5 years included studies of neuroinflammatory mechanisms and non-pharmacological treatments, such as exercise, cryotherapy, and acupuncture. These topics offer potential research directions for the future.

## Discussion

In this study, we conducted a bibliometric analysis of publications related to CIPN over the past two decades so that we could identify key hotspots and trends in CIPN research. We found that the number of publications related to CIPN research have increased rapidly since 2001. We observed a gradual increase in the number of publications related to CIPN research, especially after 2010, thus showing that this field will remain a hot topic of interest for researchers over the coming years. Our analysis showed that the United States plays an essential role in CIPN research, followed by Italy, Japan, and China; about 56.4% of all publications were published by researchers in the United States. In addition, the United States dominated our results in terms of publication output, total citations, and international collaborations. The high ranking of France and the Netherlands in terms of total citations indicates the high quality of the research activity in these countries. With regards to international cooperation, the United States, Italy, and Germany, were found to collaborate closely with other countries, while China and Japan collaborate to a lesser extent; this may be the reason for their low ranking in the number of citations. International collaboration should be strengthened in China and Japan so that higher quality publications can be disseminated.

When considering the top 10 institutions with the highest impact, we found that 70% were in the United States. The University of Milan-Bicocca from Italy contributed the highest number of publications and total citations, indicating that this institution publishes higher quality articles and could be considered for further collaboration and learning. In terms of authors, G. Cavaletti, A. A. Argyriou, S. S. Park, P. Alberti, and J. Bruna had the highest number of individual publications and all made significant contributions to the progression of CIPN research. In addition, studies by P. M. Dougherty, C. I. Loprinzi, and A. A. Argyriou had the highest average number of citations, thus indicating that these authors publish high quality research in the field of CIPN. Moreover, it is evident from the network that these teams maintain close collaboration with each other; this is why they can produce high quality publications. Our analysis showed that a number of journals are publishing CIPN-related research; the journals that have published the most articles on CIPN were *Supportive Care in Cancer, Pain, Molecular Pain*, and *Experimental Neurology*.

### Current Research Hotpots

Based on the keywords co-occurrence analysis, we were able to classify CIPN research into two key hotspots, as described below.

(I). Oxaliplatin- and Paclitaxel-Induced Peripheral Neuropathy

Neurotoxicity is commonly associated with the application of oxaliplatin and paclitaxel and therefore has gained high levels of clinical attention. Oxaliplatin-induced peripheral neuropathy (OIPN) can be classified as either acute or chronic, with a 50–90% incidence of acute neurotoxicity and a 60–80% incidence of chronic neurotoxicity (21–23). Acute OIPN usually presents with distal sensory abnormalities and dullness induced by the cold stimuli or abnormal pharyngeal sensation (24, 25). Chronic OIPN may manifest as numbness at the ends of the extremities, pain, or sensory ataxia (26). The clinical manifestations of paclitaxel-induced peripheral neurotoxicity (PIPN) are similar to those of OIPN, while 30% of patients with PIPN experience neuropathic pain (27). Large interpersonal variability exists in the intensity of the symptoms experienced by patients with OIPN and PIPN; this variability is closely related to the cumulative dose of chemotherapy, the schedule, and combination therapy (26, 28). Pre-existing risk factors, such as diabetes, hemoglobin, age, body mass index, high alcohol consumption, and genetic predisposition, are all known to increase the incidence of such diseases (29–32). In addition, the severity of CIPN has been shown to be proportional to the number of comorbidities at baseline (33). Despite recent progress, our ability to provide effective prevention and treatment strategies for CIPN remain limited. Therefore, a key direction for the future is to develop high-quality clinical research to identify effective drugs that can prevent and treat CIPN.

(II). Pain Management and QOL Issues in Patients with CIPN

Due to a lack of effective control measures, over 80% of cancer survivors experience neuropathic pain during chemotherapy. Only duloxetine is moderately recommended to relieve the chemotherapy-induced peripheral neuropathic pain (CIPNP) (8). Different types of chemotherapeutic agents can cause CIPNP and do so *via* a range of different mechanisms, such as mitochondrial damage, increased ion channel activity, neuronal injury, and inflammation, and oxidative stress (34, 35). Although chemotherapeutic agents exhibit different pathophysiology profiles, they also have overlapping contributions to the pathogenesis of neuropathic pain and may be targets for the prevention or treatment. Pain management in post-chemotherapy patients is an issue that has yet to be resolved. However, current treatment strategies have been associated with poor levels of clinical efficacy. Subsequent studies should be based on mechanisms rather than symptoms to obtain more evidence for treatment. Notably, the application of analgesic drugs must not compromise antitumor efficacy and increase the incidence of other adverse effects. Therefore, monotherapy is still recommended for the management of CIPNP (35). In addition, CIPN increases the burden on patients and has a significant and long-term impact on patient QOL (36–38). Patients with severe CIPN are more likely to experience fatigue, depression, and anxiety (5, 39). Therefore, QOL in cancer survivors cannot be ignored during the treatment and care, making this a significant topic for long-term research.

### Future Frontiers

Based on our review of the timeline of research topics for CIPN prior to 2021, we forecast that two research topics will play significant roles in the future, as follows.

(I). Study of Neuroinflammatory Mechanisms

Peripheral neuropathies are characterized by the axonal degeneration and nerve damage. Neuroinflammation has been proven to be associated with a variety of neuropathic pain models (e.g., diabetic neuropathy and traumatic nerve injury) (40, 41). Chemotherapeutic drugs can accumulate in nerve axons and dorsal root ganglia, which, in addition to activation of the immune system and immune-like glial cells (such as satellite glial cells and Schwann cells), may lead to neuroinflammation and thus to the development of CIPN (42, 43). In addition, the involvement of neuroinflammation in the pathological process of CIPN has been demonstrated in the central nervous system (44, 45). The features and mechanisms of the neuroinflammatory process differ and are dependent on the type of chemotherapeutic agents. In summary, a better understanding of the mechanisms of neuroinflammation may help us to develop useful future strategies to improve the management of CIPN. Notably, recent studies have found that ganglioside-monosialic acid (GM1), a neuroprotective agent, is effective in preventing the neurotoxicity during chemotherapy. A high-quality randomized controlled trial in colorectal cancer found that GM1 significantly reduced the incidence and severity of PIPN reported by subjects (46). Another study on breast cancer reported similar results (47). These studies may be a significant advance in neurotoxic intervention strategies for paclitaxel drugs.

(II). Non-pharmacological Interventions for CIPN

Although many drugs and preparations have been proven to exert neuroprotective effects in basic research, most of these drugs lack the support of clinical research data or have been proven to be invalid by phase III clinical studies, such as calcium and magnesium (48–50). Therefore, researchers have increasingly focused on non-pharmacological interventions, such as acupuncture, exercise, and herbal medicine. Acupuncture has shown a positive effect on CIPN, but heterogeneous design, and variability in outcome measures make it difficult to evaluate its impact (51). Notably, compared with invasive acupuncture treatment, transcutaneous electrical nerve stimulation (TENS), an inexpensive, non-invasive, and self-administered technique, is being intensively investigated as a possible adjunct to pharmacological treatment of CIPN (52, 53). Furthermore, a meta-analysis of exercise interventions reported potentially positive treatment effects (54). Combined exercise protocols (sensorimotor, endurance, and strength training) appear to greater levels of improvement in patients with CIPN (55). However, the quality of evidence and strength of recommendation put forward by these clinical reports were compromised by shortcomings in study design, sample size, and assessment methods. As a result, these promising therapies have not been recommended for the prevention and treatment of CIPN (49). Further investigation is now needed to increase the credibility and reliability of such research. Larger sample, multicenter randomized controlled studies need to be considered in future study protocols to confirm the efficacy and clarify risks.

This study has some limitations that need to be considered. First, due to software limitations, we were unable to conduct a cross-check with multiple high-quality databases, such as Pubmed and Embase; this may have led to an incomplete selection of literature. Second, the dynamic update of the databases caused a certain lag in our literature screening. Finally, due to the relatively large number of publications, some papers may have been omitted by manual screening.

## Conclusions

Our bibliometric analysis of the literature identified 2,188 publications related to CIPN between 2001 and 2021. Our analysis identified specific countries, institutions, authors, and journals, that made significant contributions to this field of research during the study period. We focused on specific research topics to explore the trends in CIPN research. Oxaliplatin, paclitaxel, QOL, and pain management, remain the current research targets in the CIPN field. Neuroinflammation provides a potential direction for the study of CIPN mechanisms. Non-pharmacological therapies, such as exercise and acupuncture therapy, will become a key research focus in the future to identify the possible beneficial treatment combinations.

## Data Availability Statement

The original contributions presented in the study are included in the article/Supplementary Material, further inquiries can be directed to the corresponding author/s.

## Author Contributions

JG, MH, and GW contributed to concept, design, and manuscript writing. ZG and LL were responsible for literature search, data acquisition, data analysis, and statistical analysis. CH and JY contributed to data acquisition, supervision, and edited the manuscript. JH and YJ reviewed the manuscript. All authors read and approved the final manuscript.

## Funding

This research was supported by the National Natural Science Foundation of China (Reference: 82004339), the Project of National Clinical Research Base of Traditional Chinese Medicine in Jiangsu Province (Reference: JD2019SZXYB02), the Jiangsu Province TCM Leading Talent Training project (Reference: SLJ0211), the Jiangsu Science and Technology Department Social Development-Clinical Frontier Technology (Reference: BE2019767 and BRA2019100), and the Scientific Research Project of Jiangsu Provincial Health Commission (Reference: H2019095).

## Conflict of Interest

The authors declare that the research was conducted in the absence of any commercial or financial relationships that could be construed as a potential conflict of interest.

## Publisher's Note

All claims expressed in this article are solely those of the authors and do not necessarily represent those of their affiliated organizations, or those of the publisher, the editors and the reviewers. Any product that may be evaluated in this article, or claim that may be made by its manufacturer, is not guaranteed or endorsed by the publisher.

## Supplementary Material

Supplementary Table 1 Details of search strategy.

Supplementary Figure 1 The collaborative relationships between different authors.
