# Supplementary material for: Bibliometric Analysis Reveals a 20-Year Research Trend for Chemotherapy-Induced Peripheral Neuropathy
Source: Front Neurol. 2022 Feb 8;12:793663. doi: 10.3389/fneur.2021.793663 (PMC8860827; doi:10.3389/fneur.2021.793663)
Supplement: Supplementary Table 1 — Details of search strategy. [file Data_Sheet_1.PDF]

Table 1. Details of search strategy.

Number	Search term
1	TS=(chemotherapy)
2	TS=(antineoplastic agents)
3	TS=(oxaliplatin)
4	TS=(paclitaxel)
5	TS=(docetaxel)
6	TS=(vinorelbine)
7	TS=(bortezomib)
8	TS=(vinc*)
9	TS=(cisplatin)
10	TS=(taxane)
11	TS=(neurotoxicity)
12	TS=(neuropa*)
13	TS=(nerve)
14	1 OR 2 OR3 OR 4 OR 5 OR 6 OR 7 OR 8 OR 9 OR 10
15	11 OR 12 OR 13
16	14 AND 15

TS = title, abstract, author keywords and keywords plus.

* = any ending to the word.
