# Supplementary figures and images for: Bibliometric Analysis Reveals a 20-Year Research Trend for Chemotherapy-Induced Peripheral Neuropathy
Source: Front Neurol. 2022 Feb 8;12:793663. doi: 10.3389/fneur.2021.793663 (PMC8860827; doi:10.3389/fneur.2021.793663)

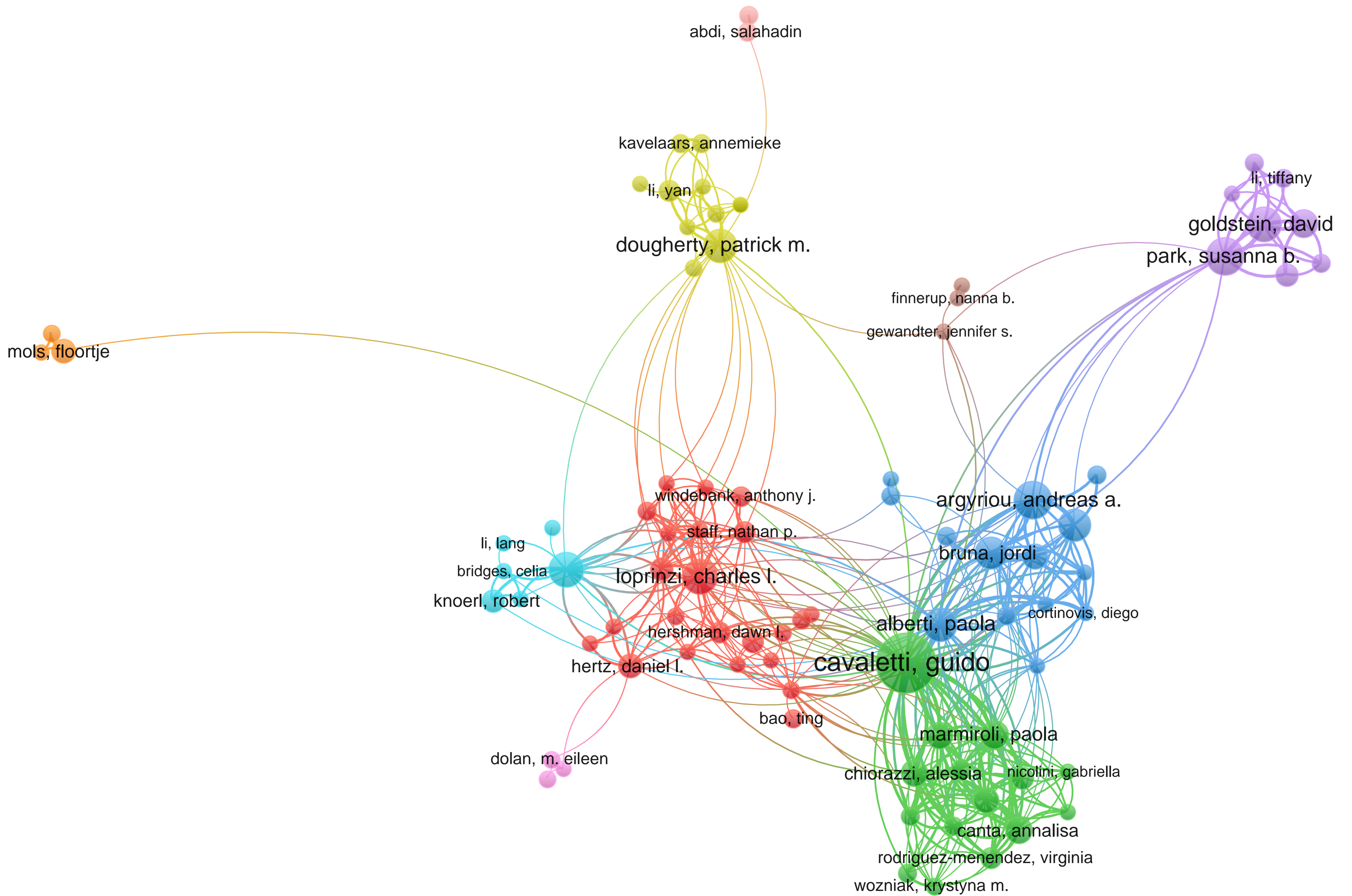

Supplement: Supplementary Figure 1 — The collaborative relationships between different authors. [file Data_Sheet_2.PDF]
